# Patient Satisfaction with Primary Health Care – A Comparison between the Insured and Non-Insured under the National Health Insurance Policy in Ghana

**DOI:** 10.5539/gjhs.v6n4p9

**Published:** 2014-04-01

**Authors:** Ama P. Fenny, Ulrika Enemark, Felix A. Asante, Kristian S. Hansen

**Affiliations:** 1 Institut for Folkesundhed, Aarhus University, Århus C, Denmark; 2 Economics Division, ISSER, University of Ghana, Accra, Ghana; 3 Department of Global Health and Development, Faculty of Public Health and Policy, London School of Hygiene and Tropical Medicine, London, UK

**Keywords:** quality of care, health insurance, patient satisfaction, Ghana, National Health Insurance Scheme

## Abstract

Ghana has initiated various health sector reforms over the past decades aimed at strengthening institutions, improving the overall health system and increasing access to healthcare services by all groups of people. The National Health Insurance Scheme (NHIS) instituted in 2005, is an innovative system aimed at making health care more accessible to people who need it. Currently, there is a growing amount of concern about the capacity of the NHIS to make quality health care accessible to its clients. A number of studies have concentrated on the effect of health insurance status on demand for health services, but have been quiet on supply side issues. The main aim of this study is to examine the overall satisfaction with health care among the insured and uninsured under the NHIS. The second aim is to explore the relations between overall satisfaction and socio-demographic characteristics, health insurance and the various dimensions of quality of care.

This study employs logistic regression using household survey data in three districts in Ghana covering the 3 ecological zones (coastal, forest and savannah). It identifies the service quality factors that are important to patients’ satisfaction and examines their links to their health insurance status.

The results indicate that a higher proportion of insured patients are satisfied with the overall quality of care compared to the uninsured. The key predictors of overall satisfaction are waiting time, friendliness of staff and satisfaction of the consultation process. These results highlight the importance of interpersonal care in health care facilities. Feedback from patients’ perception of health services and satisfaction surveys improve the quality of care provided and therefore effort must be made to include these findings in future health policies.

## 1. Introduction

Healthcare financing, one of the major channels to access to health care in developing countries, has gone through many reforms in Ghana. Past reforms in the health sector resulted in inequities in access to basic primary healthcare leading to the introduction of the National Health Insurance Scheme (NHIS) in 2005. The overriding aim of this institutional intervention is to reduce financial barriers to utilization of healthcare by reducing direct payments for services at health facilities. This is intended to narrow the inequalities engendered by the previous system which relied mainly on user fees. Ghana has joined the ranks of a number of developed countries such as Germany who have implemented this form of social health insurance in a bid to ensure access to care. The current efforts world-wide is to move from systems that depend mainly on user fees to prepayment and risk pooling (WHO, 2010).

Since its introduction, there have been increasing levels of utilisation of health services as had been anticipated with the implementation of the scheme (Witter & Garshong, 2009; NDPC, 2009). However, this has put a strain on both the health workers as there has been limited improvement in infrastructure and staffing levels (MOH, 2007a; Turkson, 2009; SEND, 2010). Fundamentally, an increase in demand for health care suggests an increase in supply of services by expanding available inputs such as equipments, trained staff and consultation rooms. Failure to do so will result in health facilities relying on old equipment and same level of staffs who are likely to be overwhelmed with the added workload. Patient dissatisfaction with poor quality service is likely to affect their decisions to remain enrolled in the NHIS which ultimately makes the scheme less attractive to new members. Therefore while removing financial barriers to improve access to care; it is also important that attention is given to the quality of care provided. For the sustainability of the NHIS, continuous feedback is needed to improve the quality of service provided

Prior to this study, there were assertions that the insured were unsatisfied with treatment offered them at health facilities. They faced longer queues at health facilities and therefore endured longer waiting times mainly due to the administrative bottlenecks. Therefore, one of the aims of this study is to first determine the levels of patient satisfaction with the quality of care at the primary care level and examine any differences in service experiences between insured and non-insured members. The second objective is to establish the key factors that influence patients’ overall satisfaction with health care.

Clearly, there is the need to assure people of access to good quality care since providing services at an unacceptable low quality and with high indirect costs of access defeats the whole aim of the NHIS. These results can subsequently provide information for developing future health policies.

### 1.1 Healthcare, Health Insurance and Quality of Care in Ghana

#### 1.1.1 Overview of the Health Sector in Ghana

Health care delivery in Ghana is provided by both the public and private (private-for-profit and private-not-for-profit) sectors, with the public sector organized according to national (teaching hospitals), regional (regional hospitals), district (district hospitals), sub-district (health centres) and community levels (CHPS). Community-based Health Planning and Services (CHPS) is a programme for transforming clinic based primary health care to community-based health services. In spite of this, service delivery is beset with staff shortages, limited funds often due to untimely release of reimbursed funds from the National Health Insurance Authority (NHIA) and government as well as ageing equipment (MOH, 2007a).

The organisation of the health system is hierarchical, moving from the national level down to community levels. It incorporates a referral system from lower levels to the level immediately above them and s supervisory system from the upper to lower levels. Sub-district (health centres) and community levels (CHPS) provide primary care and are often manned by medical assistant or a nurse. While district and regional hospitals, provide both secondary health care as well as primary health care. District hospitals are staffed with one or more qualified medical doctors, nurses, pharmacists, laboratory technicians, auxiliary nurses and other support personnel. The tertiary services including specialised clinical care are provided at the teaching hospitals. However, weak health systems at the primary level and a weak referral system mean that many individuals visit secondary and tertiary levels for outpatient care.

The Ghana Health Service as part of its drive to make healthcare more accessible to people and improve quality of care provided put together a policy document on quality assurance. A five year Quality Assurance Strategic Plan (2007-2011) was developed to ensure delivery of patient-centred, safe and quality clinical care. The guiding principles of the plan include: improve client-focused services; improve patient safety; improve clinical practice and improve management systems and accountability (GHS, 2007). The report points to the fact that adherence to quality of care guidelines stipulated by healthcare providers have been very often short of satisfactory. Providers are perceived by the clients (users) to be unresponsive to their needs. Providers on the other hand, describe their working conditions to be poor due to the lack of equipment and essential drugs and supplies. The health service system is also characterised by poor gate keeping due to a weak referral system making it difficult to coordinate processes within health facilities in any given district.

#### 1.1.2 Health Insurance in Ghana

The National Health Insurance Act, 2003 (Act 650) established the National Health Insurance Scheme (NHIS) with the aim of increasing access to health care and improving the quality of basic health care services for all citizens, especially the poor and vulnerable. The law establishing the scheme allows for the concurrently operation of District-Wide (Public) Mutual Health Insurance schemes, Private Mutual Health Insurance schemes and Private Commercial Health Insurance schemes. The initially defined benefit package under the scheme includes inpatient hospital care, outpatient care at primary and secondary levels, and emergency and transfer services. The benefit package covers about 95% of diseases in Ghana such as malaria, cervical and breast cancer, surgical operations, physiotherapy, maternity care (antenatal, post natal, deliveries), dental care and eye care. Each district mutual health insurance scheme also uses its discretion to determine additional benefits the scheme could provide. The NHIS scheme has an exemption policy to ensure that the poor and other vulnerable groups have access to healthcare. The exempt groups are children under the age of 18 years, the elderly above the age of 70 and the indigent (poor).

The introduction of the NHIS has led to an increase in utilisation of health services which requires increased inputs but it is unclear how the supply side was strengthened to cope with this. Some regions are faced with a reduction in health workers but considerable increase in the number of insured. For instance the in the Northern region, the number of doctors reduced from 32 to 26 between 2006 and 2008 while NHIS membership increased from 281,775 to 828,805 (SEND, 2010). There is also anecdotal evidence that clients paying user fees enjoy better healthcare by providers. However, the Citizen’s Assessment report (2008) indicate that about half (50%) of the respondents in both the household survey and patient exit survey perceive NHIS card-holders to receive better health care services than non-card holders, while only about 20% think otherwise.

One of the core functions of the NHIS scheme is to ensure that quality care is provided to its members. In order to achieve that, the Scheme has an accreditation programme. It runs a formal accreditation process whereby all facilities whether private or public are inspected to ensure they meet pre-determined criteria before being granted accreditation (NHIA, 2010). It also stipulates post-accreditation monitoring to ensure that medical procedure and the administration of drugs are appropriate and comply with the accepted medical practice and ethics. Quality of care provided at facilities is important given the fact that it plays two essential roles; first it encourages the insured members to remain insured as they enjoy quality services and secondly encourages new members to enrol in the scheme. In the absence of these the sustainability of the scheme remains questionable.

### 1.2 Defining Quality of Care and Conceptual Framework

#### 1.2.1 Defining and Measuring Quality of Care

Quality of care is often very difficult to define and measure. The complex nature of healthcare and the various players with their varying degrees of interest within each group adds to this complexity (Donabedian, 1980; Pettersen et al., 2004; Harteloh, 2004; Naveh & Stern, 2005; McLaughlin & Kaluzny, 2006; Ladhari, 2009). As a result there is a need for a definition that is multi-dimensional to capture all differing perceptions of what encompasses quality of care. In principle, there is an agreement that if patients can get the services they need and if these services provided are beneficial then quality of care is assured. Therefore, the Institute of Medicine defines quality of care as “the degree to which health services for populations increase the likelihood of desired health outcomes and are consistent with current professional knowledge” (IOM, 2001).

Measuring quality of care follows the same path of difficulty as its definition. Some of the frameworks for quality of care assessment include the World Health Organisation (WHO) quality of care framework, the Bamako Initiative and some of the more disaggregated approaches (Donabedian, 1980; Maxwell, 1984; HSRG, 1992; Winefield, Murrell, & Clifford, 1995). The WHO quality of care assessment is based on three intrinsic goals which include optimal health, responsiveness and fairness in financing (Evans et al., 2001). The Bamako Initiative was defined around the mid-1980s and was more economic oriented but the underlying goal was to achieve quality of care. The components included effectiveness, efficiency, sustainability and equity (Knippenberg et al., 1997). The disaggregated frameworks agree on the complexity and multidimensionality of quality of care and therefore construct several dimensions or components which explain their definition of quality (Campbell et al., 2000).

**Figure 1:**
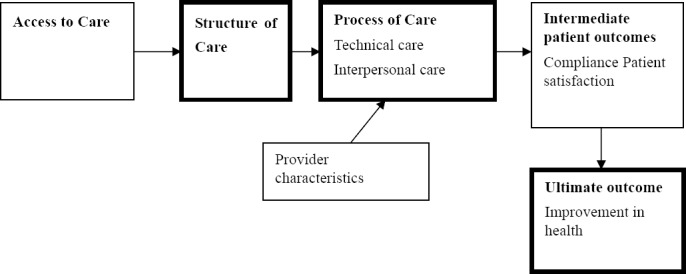
Donabedian’s structure-process-outcome paradigm Source: Donabedian, 1980

This study adopts the Donabedian Model which has been widely recognized and applied in many health care related fields (Donabedian 1980; 1986; and 1988). The framework divides factors impacting quality into structures, processes and outcomes, connected by unidirectional arrows in that order (Figure 1). Structure refers to material characteristics and resources of the providers of health care; process refers to the interaction between providers and their patients; and finally outcomes which can be measured as health status, deaths or disability-adjusted life years (Peabody et al., 2005). The model is however criticised for its sequential and linear progression from structure to process and outcome. Some suggest that there are other critical factors such as patient characteristics and environmental factors that need to be incorporated for a more holistic evaluation of quality care (Mitchell et al., 1998; Carayon et al., 2006; Coyle et al., 1999). Donabedian reiterates that the three spheres have within them linkages that allow researchers to develop conceptual frameworks for understanding their own health systems (Donabedian, 2003). Table 1 depicts a framework of indicators for evaluating patient satisfaction adapted from the Donabedian model.

**Table 1:** Framework of indicators for patient satisfaction

Variable	Measure
**Structure Variables**	Consulted with a trained personnel
	Received medicines
**Process Variables**	Overall assessment of the friendliness of staff
	Overall assessment of waiting time in facility
	Satisfaction at
	(i) the reception
	(ii) information department (records)
	(iii) consultation
	(iv) laboratory/x-ray
	(v) pharmacy/dispensary
**Outcome Variable**	Overall satisfaction with quality of care received from facility

#### 1.2.2 Patient Satisfaction

Patient satisfaction is often associated with positive emotions drawn from interactions with health service providers. Some predictive factors of patient satisfaction relate to caring, empathy, dependability and responsiveness of providers (Tucker & Adams, 2001). Fowdar (2005) includes dimensions such as core services; customization; professional credibility; competence; and communications in the measurement of patient satisfaction. Based on the Donabedian model, healthcare quality can also be categorized in three ways looking at amenities in the health facility (structure) technical aspects and interpersonal care as part of the process component and how these affect intermediate outcomes such as compliance and patient satisfaction and finally the ultimate outcome of health improvement (Donabedian, 1986).

Patient satisfaction is seen as both an outcome and an indicator and may offer a simple first step into understanding quality of care. Some authors believe that studying healthcare quality from the patient’s perspective provide valid and unique information about the quality of care (Ware & Stewart, 1992). A number of studies have used patient perspectives as a key measure of evaluating healthcare quality (Boyer et al., 2006; Aditi, 2009; Sodani, 2010; Atinga; 2011). Patients’ satisfaction with health services has been shown to improve patients’ quality of life (Dagger & Sweeney, 2006). Notably, dissatisfied patients are less likely to follow instructions for taking medications or may not attend follow-up care or discourage family members and friends from seeking health care services. However, some authors are of the opinion that patients lack the ability to assess the technical aspect of care which often encompasses broader healthcare quality measures, such as financial performance, logistics and staff competence (Eiriz & Figueiredo, 2005). These studies confirm the complexity of capturing patient satisfaction given the vast number of factors that affect individual satisfaction; and stress the error of using one measure or a group of variables to measure quality of care (Zastowny et al., 1995; Debono, 2009). However, feedback from patient satisfaction surveys is useful to improvements in quality of care (Debono, 2009).

## 2. Methods

### 2.1 Household Survey

Ghana is divided into 10 administrative regions which are subdivided into 170 districts. The administrative districts cut across 3 agro-ecological zones namely coastal, forest and savannah. A district was selected in each zone making a total of 3 districts surveyed. The sampling frame was the Enumeration Areas (EAs) from the 2000 Ghana Population and Housing Census for the selected districts. A total of 27 EAs which were representative of each district were selected. Subsequently, 30 households were systematically sampled from the household listing in each EA to obtain the required sample size giving a total of 2430 households in all three districts. The household questionnaire was administered to the head of household between January and April, 2011. For each household, data was collected on individual and household characteristics, income, education, health insurance status, and treatment seeking behaviour, dimensions of quality of care, choice of provider and community characteristics.

### 2.2 Data Analysis

#### 2.2.1 Conceptual Framework

Based on the Donabedian “structure-process-outcome” trilogy for assessing patient satisfaction, we construct a framework to demonstrate the various links to the various dimensions of quality of care (Table 1). Patient satisfaction is an interplay of service quality, expectations, emotions experienced at time of service delivery and many other unobservable factors. This framework allows insight into patient satisfaction at the various level of treatment. Some studies have shown a clear link between patient satisfaction and a variety of explanatory factors, among which service quality has been important (Rao et al., 2006; Zineldin, 2006). On the structure dimension we hypothesis that patients who consult with trained personnel at the health facilities and the availability of medicines improve overall satisfaction with quality of care. Rao et al. (2006) using a cross-sectional survey of health facilities and patients in clinics in India indicate that for outpatients, doctor behaviour had the largest effect on general patient satisfaction followed by medicine availability, hospital infrastructure, staff behaviour, and medical information. Patient satisfaction with availability, quantity and quality of drugs has been shown to be an important determinant of satisfaction and utilization of health services (Haddad & Fournier, 1995; Balthusen & Yaomeze, 2006; Mamdani & Bangser, 2004). Balthusen and Yaomeze (2006) report dissatisfaction among users and non-users with regards to the availability of drugs.

The process dimension includes perceived manners of providers; overall satisfaction is enhanced when staff are friendly and polite. We extend this dimension to cover patient-provider interactions at the various points of service at the health facilities (reception, records, consultation, laboratory and pharmacy). We hypothesise that good support from personnel in these areas of service will improve overall satisfaction with care. Also, patient satisfaction with waiting time will influence positively their overall satisfaction with care. A number of studies show that delays encountered at health facilities greatly influenced dissatisfaction with overall care (Barlow, 2002; Bielen & Demoulin, 2007; Duggirala et al., 2008). The influence of individual characteristics such age, gender and education on patient satisfaction have been inconclusive and contradictory (Tucker, 2002) but they are included as possible explanatory variables in this study.

#### 2.2.2 Statistical Analysis

The analysis is restricted to the perceptions of patients who chose a formal care provider once the decision to seek care had been made. In the household survey, the quality of care dimensions were asked using a 4-scale measure (not satisfied, somewhat satisfied, satisfied and very satisfied). First, the study uses Kruskal-Wallis rank test and Pearson χ^2^ test to assess whether the differences between the insured and non-insured are statistically significant. We estimated relationships between process measures and patient satisfaction using logistic regression model with “overall quality of care received” as a dependant variable and process-of-care measures as the independent variables. We extended the model to include individual characteristics including age, sex, education, insurance status as well as the type of provider chosen (regional/district hospital, public health centre or private clinics). Age is captured through dummy variables: less than 18 years, 18-69 years and 70 years and above. This set up reflects the various age levels used by the NHIS scheme to grant exemptions from premiums. Health insurance status was obtained through a question asking if the individual had a valid NHIS card or not. Typically, the structure variables should have been added as possible explanatory variables but every patient that sought care in the facility consulted with trained personnel and received the necessary drugs prescribed at the facility. STATA 11 was used for all statistical analysis (StataCorp, 2009).

## 3. Results

### 3.1 Descriptive Summary Statistics

Table 2 presents the percentage distribution of the personal and household attributes of the insured and non-insured groups. In total, 11,089 individuals of 2,430 households were available for the analysis in the survey data. Of these households, 39% were insured and 61% uninsured. A total of 1,013 sought care after reporting illness and out of this 716 used either public or private health facilities. A summary of the choice of provider by gender, health insurance status, age, educational level and nature of illness is presented in Appendix 1.

**Table 2:** Basic statistics of household attributes

Variable Personal characteristics	Health Insurance Status			

	Insured		Uninsured	
	Mean	Std. Dev.	Mean	Std. Dev.
**Sex**				
Male	0.447	0.497	0.506	0.500
Female	0.553	0.497	0.494	0.500
**Age**				
mean	24.692	21.158	22.820	18.686
<18 years	0.498	0.500	0.495	0.500
18-69 years	0.450	0.498	0.482	0.500
≥70 years	0.052	0.223	0.024	0.153
**Education**				
No education	0.284	0.451	0.381	0.486
Some primary	0.383	0.486	0.363	0.481
Completed primary	0.244	0.430	0.210	0.407
Secondary or higher	0.088	0.284	0.046	0.210
**Employment(≥15 years)**				
Formal	0.096	0.294	0.046	0.210
Informal	0.904	0.294	0.954	0.210
**Household characteristics**				
Household Size	4.318	2.575	4.768	2.747
% with female head	0.244	0.429	0.171	0.377
**Community**	0.641	0.479	0.498	0.500
% with health facility in community				
**N**	**4,311**		**6,778**	

Source: Household data January to April, 2011.

### 3.2 Overall Satisfaction with Quality of Care Received

Survey respondents who received care in the last 4 weeks (N= 716), were asked to rate their overall satisfaction with the quality of care they received. Overall, 36% of them were very satisfied with the overall care; 53% were satisfied; 9% were somewhat satisfied and 2% were not satisfied. In the insured group 38% were very satisfied; 53% were satisfied; 8% were somewhat satisfied and 1% were not satisfied. In the uninsured on the other hand, 33% were very satisfied; 53% were satisfied; 10% were somewhat satisfied and 3% were not satisfied. Across facility types, for the regional/district hospitals 35% of respondents were very satisfied with overall care. Majority of those who went to private hospitals/clinics were very satisfied with overall care (51%) and none were dissatisfied; this is significant at the 1% level (Table 3).

**Table 3:** Number of patients (%) who were satisfied with overall care

	N	Very satisfied %	Satisfied %	Somewhat satisfied %	Not satisfied %
**Total**	716	36.4	53.2	8.9	1.5
**Insurance Status**					
**Insured**	519	37.6	53.2	8.3	0.09
**Uninsured**	197	33.2	53.4	10.4	3.0
*p* *= 0.131 χ^2^= 5.623					
**Facility type**					
**Regional/District Hospitals**	322	35.1	50.6	12.4	1.9
**Private hospital/clinic**	107	51.4	42.1	6.5	0
**Health centres/CHPS**	287	30.3	58.5	9.4	1.7
p* = 0.001 χ^2^= 16.770					

p* Kruskal-Wallis rank test.

Source: Household data January to April, 2011.

### 3.3 Overall Satisfaction by Dimensions of Quality of Care and Health Insurance Status

Overall, both groups were very satisfied with the consultation process (Table 4). A higher proportion of insured persons were very satisfied with waiting time compared to the uninsured (21% vs. 19%) and very satisfied at the laboratory/x-ray department (27% vs. 22%). We also see differences in the satisfaction levels reported at the pharmacy/dispensary among the two groups. In the insured group, 29% were not satisfied with the services rendered at the pharmacy/dispensary compared to 8% in the uninsured group. This is statistically significant at 10% level. At this level of significance the association is quite moderate.

**Table 4:** Number of patients (%) who were satisfied with care by quality of care dimensions (Insured vs. the Uninsured)

Quality of Care Variables	N	Very satisfied (%)	Satisfied (%)	Somewhat satisfied (%)	Not satisfied (%)
**Waiting time**					
**Insured**	517	21.3	52.6	19.3	6.8
**Uninsured**	193	19.2	51.8	21.8	7.3
p* = 0.842 χ^2^= 0.831					
**Friendliness of staff**					
**Insured**	518	32.8	57.5	7.9	1.7
**Uninsured**	193	32.5	54.1	11.3	2.1
p* = 0.515 χ^2^= 2.286					
**Satisfaction at reception**					
**Insured**	517	23.8	52.3	20.7	3.1
**Uninsured**	191	25.1	55.5	11.1	3.1
p* = 0.619 χ^2^= 1.782					
**Satisfaction at records department**					
**Insured**	513	25.9	55.6	14.4	4.1
**Uninsured**	193	25.9	58.5	11.4	4.1
p* = 0.669 χ^2^= 1.557					
**Satisfaction with consultation**					
**Insured**	516	60.2	34.0	4.3	1.6
**Uninsured**	191	60.2	32.5	3.7	3.7
p* = 0.367 χ^2^= 3.168					
**Satisfaction at laboratory/x-ray**					
**Insured**	170	26.5	58.2	11.8	3.5
**Uninsured**	55	21.8	63.6	10.9	3.6
p* = 0.900 χ^2^= 0.584					
**Satisfaction at pharmacy/dispensary**					
**Insured**	511	8.4	22.2	40	29.4
**Uninsured**	191	25.7	48.2	22	4.2
p* = 0.094 χ^2^= 6.393					

p* Kruskal-Wallis rank test.

Source: Household data January to April, 2011.

### 3.4 Logistic Regression Analysis

Results from the regression model (see Table 5), show three influential factors at 5% significant level (satisfaction with consultation, satisfaction with waiting time and satisfaction with friendliness of staff). Patients satisfied with consultation were 1.8 times more likely to report satisfaction with overall quality of care than those dissatisfied, *p*-value = 0.001. Patients who were satisfied with friendliness of staff were 3 times more likely to report satisfaction with overall quality of care than those dissatisfied, *p*-value = 0.000. Also, patients who were satisfied with the waiting time were 0.8 times less likely to report satisfaction with overall care than those dissatisfied (*p* = 0.05).

**Table 5:** Logistic regression with selected independent variables

Variable	Odds Ratio	*p* - value	95% confidence	
**Insured**	0.43	0.30	-0.38	1.25
**Age**				
**18-69 years^ζ^**	-0.24	0.64	-1.26	0.77
**≥70 years**	-0.31	0.67	-1.72	1.10
Education Some primary*	-0.96	0.09	-2.07	0.16
**JSS/Middle**	0.71	0.89	-0.93	1.06
**Secondary & above**	0.50	0.47	-0.86	1.86
**Facility**				
**Public health centre^δ^**	-0.87	0.25	-2.32	0.59
**Private hospital/clinic**	0.25	0.54	-0.55	1.05
**Quality of process care**				
**Satisfaction at reception**	0.13	0.76	-0.74	1.00
**Satisfaction at records department**	0.60	0.17	-0.26	1.46
**Satisfaction with consultation**	1.76	0.001	0.69	2.82
**Satisfaction at pharmacy/dispensary**	0.12	0.77	-0.67	0.91
**Satisfaction with waiting time**	0.84	0.05	-0.01	1.68
**Satisfaction with friendliness of staff**	3.02	0.000	2.19	3.85

ζ Omitted variable – < 18 years.

* Omitted variable – no education.

δ Omitted variable – Regional/district hospital.

Source: Household data January to April, 2011.

## 4. Discussions

Overall, majority of patients are generally satisfied with the health facilities they chose and there were no significant differences between both groups in overall satisfaction ratings. More than half of the patients who sought care at private hospitals or clinics rated their overall satisfaction as “very good” compared to 35% and 30% of those who attended regional/district hospitals and public health centres respectively. Since the study did not incorporate specific provider attributes it will be difficult to give any conclusive explanations for this finding.

Going by the disaggregated approach adapted from the Donabedian structure-process-model, the results show no significant differences between the insured and uninsured groups with regards to the perception of friendliness of medical staff, satisfaction at the reception, records departments and in consultation rooms. However, pharmacy/dispensaries received the worst ratings among the insured compared to the uninsured and this difference is moderately significant. Dalinjong and Laar (2012) conducted a client and provider perceptions study under the NHIS in two districts in the northern part of Ghana using cross sectional data. The major complaint by the insured patients in their study was the longer waiting times they faced while receiving their drugs.

Using logistic regression, the results demonstrate that the three predictors of overall satisfaction with quality of care are satisfaction with consultation, satisfaction with waiting time and satisfaction with friendliness of staff. We hypothesised that there would be positive association between specific care processes and overall satisfaction perceptions. Clearly, interpersonal relationships hold the key to patient satisfaction. This is also confirmed by findings from other client satisfaction surveys. The importance of politeness, friendliness and respectful attitudes of health staff to overall satisfaction with care has been documented (Aldana et al., 2001; De Allegri et al., 2006c). For instance, Atinga (2011) looks at the service quality dimensions of a number of NHIS accredited facilities and concludes that interaction with service providers and demeanour of care givers were given high ratings among the patients surveyed. The socio-demographic characteristic variables (age and education) and being insured on the other hand were not significantly related to overall satisfaction. Some authors suggest that how patients are treated at health facilities outweighs their perception of quality than lowering costs (Wilson et al., 2007; Volpp, 2007).

Some limitations arise in a study of this nature where subjectivity of the some of the variables poses a challenge. Different people may rate the same services differently and can also be influenced by their individual expectations and characteristics. For instance, the severity of an illness may increase the quality provided by the medical personnel which will trigger higher ratings of patient satisfaction. In other cases, patients may also be judging services against very low expectations. Beyond this, patients’ social and cultural beliefs could be fundamental to their perception of quality of care received; factors which have not been captured in our model. Finally, this approach potentially leaves out people who were dissatisfied with the service and no longer use the facilities which might have lead to an underestimation of dissatisfaction levels.

## 5. Conclusion

We believe that the Donabedian ‘‘structure–process–outcome’’ conceptual model provides a framework for assessing factors that predict patient satisfaction with quality of care. Conceptually, this is complex given the many varied attributes of quality of care. However, the results confirm the high level of satisfaction ratings given by health seekers at the household level. The fact that two of the significant factors involved interpersonal processes reveals the importance that patients place on these measures. Presumably staff attitudes play an important role in the way patients perceive quality of care and could improve patient outcomes through better treatment adherence. The fact that being insured did not influence overall satisfaction with quality of care may lay credence to the fact that lowering the direct payments at health facilities does not guarantee good quality care. However, it is important to note that the perception of quality by the insured and uninsured users may be impacted by other factors that have not been identified in this study. Also, whether attitudes of health care staff is progressively worsening with increasing utilization of services as the result of the NHIS is yet to be determined.

The highest dissatisfaction was recorded at the pharmacy/dispensary with the insured group, the least satisfied compared to the uninsured group. It is not clear what the administrative processes are encountered by the insured at the pharmacies or dispensaries. Clearly, there are some bottlenecks in this area of service especially for scheme members. Further research is needed to ascertain what these are and how they can be addressed in the future.

Quality of care is a broad concept that no single approach adequately and fully measures. Hence we cautiously point out that these results measured only one part of an overall quality evaluation effort. However, these results can contribute to our understanding of how patients perceive certain aspects of quality of care and feedback from surveys of this nature can be of considerable policy significance.

## Appendix

**Table 1:** Choice of care, conditioned on reporting illness (%)

	Regional/District hospital (%)	Private hospital/clinic (%)	Public health centre/clinic (%)	Informal care (%)	P

	N=319 (31.7)	N=106 (10.5)	N=286 (28.4)	N=295 (29.3)	
**Male**	30.16	9.28	30.39	30.16	P= 0.377
**Female**	32.99	11.51	26.8	28.69	
**Insured**	39.9	13.58	32.12	14.4	P= 0.000
**Uninsured**	19.8	6.11	22.74	51.34	
**<18**	24.49	10.53	34.55	30.43	P= 0.000
**18-69**	36.5	10.13	23.21	30.17	
**70+**	41.18	12.75	25.49	20.59	
**No education**	32.19	11.25	21.56	35	P = 0.034
**Some primary**	31	7.01	34.69	27.31	
**JSS/Middle**	36.36	10	28.18	25.45	
**Secondary & above**	32.89	10.53	26.32	30.26	
**Malaria/Fever**	24.15	11.65	35.23	28.98	P = 0.000
**Other Acute diseases**	33.26	8.62	25.46	32.65	
**Chronic diseases**	44.23	12.82	22.44	20.51	

## References

[ref1] Aldana M. J, Piechulek H, Sabir A. A (2001). Client satisfaction and quality of health care in rural Bangladesh. Bulletin of World Health Organization.

[ref2] Atinga R. A, Abekah-Nkrumah G, Domfeh K. A (2011). Managing healthcare quality in Ghana: a necessity of patient satisfaction. International Journal of Health Care Quality Assurance.

[ref3] Baltussen R, Ye Y (2006). Quality of care of modern health services as perceived by users and non-users in Burkina Faso. Int J Qual Health Care.

[ref4] Barlow G. L (2002). Auditing hospital queuing. Managerial Auditing Journal.

[ref5] Bielen F, Demoulin N (2007). Waiting time influence on the satisfaction-loyalty relationship in services. Managing Service Quality.

[ref6] Boyer L, Francois P, Doutre E, Weil G, Labarere J (2006). Perception and use of the results of patient satisfaction surveys by care providers in a French teaching hospital. Int J Qual Health Care.

[ref7] Campbell S. M, Roland M. O, Buetow S. A (2000). Defining Quality of care. Social Science and Medicine.

[ref8] Carayon P, Schoofs Hundt A, Karsh B.-T, Gurses A. P, Alvarado C. J, Smith M, Flatley Brennan P (2006). Work system design for patient safety: the SEIPS model. Quality and Safety in Health Care.

[ref9] Coyle Y. M, Battles J. B (1999). Using antecedents of medical care to develop valid quality of care measures. International journal for quality in health care : journal of the International Society for Quality in Health Care/ISQua.

[ref10] Dagger T, Sweeney J. C (2006). The effects of service evaluation on behavioral intentions and quality of life. Journal of Service Research.

[ref11] Dalinjong P. A, Laar A. S (2012). The national health insurance scheme: perceptions and experiences of health care providers and clients in two districts of Ghana. Health Economics Review.

[ref12] De Allegri M, Sanon M, Sauerborn R (2006c). “To enrol or not to enrol?” A qualitative investigation of demand for health insurance in rural West Africa. Social Science & Medicine.

[ref13] Debono (2009). Compliments and patient satisfaction: A comprehensive review of the literature. Centre for Clinical Governance Research in Health, UNSW.

[ref14] Donabedian A (1980). Explorations in Quality Assessment and Monitoring, Volume 1: The Definition of Quality and Approaches to its Assessment.

[ref15] Donabedian (1988a). The Quality of Care: How can it be Assessed?. Journal of American Medical Association.

[ref16] Donabedian (2003). An Introduction to Quality Assurance in Health Care.

[ref17] Duggirala M, Rajendran C, Anantharaman R. N (2008). Patient-perceived dimensions of total quality service in healthcare. Benchmarking: An International Journal.

[ref18] Eiriz V, Figueiredo J. A (2005). Quality evaluation in healthcare services based on customer-provider relationships. International Journal of Healthcare Quality Assurance.

[ref19] Evans D. B, Edejer T. T, Lauer J, Frenk J, Murray C. J (2001). Measuring quality: from the system to the provider. International Journal for Quality Health Care.

[ref20] Fowdar R (2005). Identifying health care attributes. Journal of Health and Human Services Administration.

[ref21] Ghana Health Service (2007). Quality Assurance Strategic Plan for Ghana Health Service (2007-2011).

[ref22] Ghana Health Service (2009). Annual Report 2009.

[ref23] GHANA NHIS (2010). GHANA NHIS Report. Balancing Access with Quality Health Care: An Assessment of the NHIS in Ghana (2004-2008).

[ref24] Haddad S, Fournier P (1995). Quality, costs and utilization of health services in developing countries. A longitudinal study in Zaire. Soc Sci Med.

[ref25] Harteloh P. P. M (2004). Understanding the Quality Concept in Health Care. Accreditation and Quality Assurance.

[ref26] Health Services Research Group (1992). Quality of care: What is quality and how can it be measured?. Can. Med. Assoc. J.

[ref27] Institute of Medicine (2001). Crossing the Quality Chasm.

[ref28] Knippenberg R, Soucat A, Oyegbite K, Sene M, Bround D, Pangu K, Alihonou E (1997). Sustainability of health care including expanded program of immunizations in Bamako Initiative programs in West Africa: an assessment of 5 years’ field experience in Benin and Guinea. International Journal of Health Planning and Management.

[ref29] Ladhari R (2009). A review of twenty years of SERVQUAL research. International Journal of Quality and Service Sciences.

[ref30] Mamdani M, Bangser M (2004). Poor people’s experiences of health services in Tanzania: a literature review. Reprod Health Matters.

[ref31] Maxwell R. J (1984). Quality assessment in health. Br. Med. J.

[ref32] McLaughlin C. P, Kaluzny A. D (2006). Continuous Quality Improvement in Health Care.

[ref33] Mitchell P. H, Ferketich S, Jennings B. M (1998). Quality health outcomes model. American Academy of Nursing Expert Panel on Quality Health Care.

[ref34] MoH (2007a). Human Resource Policies and Strategies for the Health Sector (2007-2011).

[ref35] Witter S, Koot J, Cammack T, Buckle G, Bjerrum A, MOH (2009). Independent Review Health Sector Programme of Work 2008: Pulling together, achieving more.

[ref36] Naidu A (2009). Factors affecting patient satisfaction and healthcare quality. International Journal of Health Care Quality Assurance.

[ref37] National Development Planning Commission (2009). 2008 Citizens’ Assessment of the National Health Insurance Scheme of Ghana, Towards a Sustainable Health Care Financing Arrangement that Protects the Poor.

[ref38] National Health Insurance Authority (2010). Annual Report 2009, National Health InsuranceAuthority, Accra. http://www.nhis.gov.gh/_Uploads/dbsAttachedFiles/1(1).pdf.

[ref39] Naveh E, Stern Z (2005). How quality improvement programmes can affect general hospital performance. International Journal of Healthcare Quality Assurance.

[ref40] Peabody J, Taguiwalo M, Robalino D, Frenk J, Jamison D (2005). Improving the quality of care in developing countries. Disease control priorities in developing world.

[ref41] Peterson S, Nsungwa-Sabiiti J, Were W, Nsabagasani X, Magumba G, Nambooze J, Mukasa G (2004). Coping with paediatric referral –Ugandan parents’experience. Lancet.

[ref42] Rao K. D, Peters D. H, Bandeen-Roche K (2006). Toward patient centered health services in india—a scale to measure patient perceptions of quality. International Journal for Quality in Health Care.

[ref43] Sodani P. R, Kumar R. K, Srivastava J, Sharma L (2010). Measuring patient satisfaction: A case study to improve quality of care at public health facilities. Indian J Community Med.

[ref44] StataCorp (2009). Stata Statistical Software: Release 11.

[ref45] Tucker J (2002). The moderators of patient satisfaction. Journal of Management in Medicine.

[ref46] Tucker J. III, Adams S. R (2001). Incorporating patients’ assessments of satisfaction and quality: an integrative model of patients’ evaluations of their care. Managing Service Quality.

[ref47] Turkson P. K (2009). Perceived quality of healthcare delivery in rural districts of Ghana. Ghana Medical Journal.

[ref48] Volpp K. G (2007). Designing a model health care system. American Journal of Public Health.

[ref49] Ware J. E, Stewart A, Durham N. C, Stewart A. L (1992). Measures for a new era of health assessment. Measuring Functioning and Well-Being.

[ref50] Wilson I. B, Landon B. E, Marsden P. V, Hirschhorn L. R, McInnes K, Ding L, Cleary P (2007). D Correlations among measures of quality in HIV care in the United States: cross sectional study. British Medical Journal.

[ref51] Winefield H. R, Murrell T. G, Clifford J (1995). Process and outcomes in general practice consultations: Problems in defining high quality care. Soc. Sci. Med.

[ref52] Witter S, Garshong B (2009). Something old or something new? Social health insurance in Ghana. BMC International Health and Human Rights.

[ref53] World Health Organisation (2010). The World Health Report 2010: Health Systems Financing: The path to universal coverage. 2010. http://www.who.int/whr/2010/10.

[ref54] Zastowny T. R, Stratmann W. C, Adams E. H, Fox M. L (1995). Patient satisfaction and experience with health services and quality of care. Qual Manag Health Care.

[ref55] Zineldin M (2006). The quality of health care and patient satisfaction: an exploratory investigation of the 5Qs model at some Egyptian and Jordanian medical clinics. International Journal for Quality in Health Care.

